# Massive Ovarian Oedema in Pregnancy: A Diagnostic Dilemma

**DOI:** 10.7759/cureus.81640

**Published:** 2025-04-03

**Authors:** Jaice M Devasia, Bharathi D Rao

**Affiliations:** 1 Obstetrics and Gynecology, Kasturba Medical College, Manipal Academy of Higher Education, Mangalore, IND; 2 Obstetrics and Gynecology, Whittington Health NHS Trust, London, GBR

**Keywords:** exploratory laparotomy, histology, neoplasm, ovarian oedema, pregnancy

## Abstract

Massive oedema of the ovary is a rare clinical entity and poses a clinical challenge due to the potential for misdiagnosis. This is a case of a 27-year-old pregnant woman with incidentally detected ovarian oedema on routine dating scan. She was asymptomatic. Clinical examination revealed a midline pelvic mass, which was firm to hard in consistency, corresponding to a 14-week size of a gravid uterus. After medical termination as requested by the patient, further imaging was done. Contrast-enhanced CT showed a well-defined cystic lesion in the left adnexa with a thick, irregular enhancing wall and multiple intensely enhancing tortuous tufts of vessels seen from the ovarian artery and draining into the left ovarian vein, likely of old ovarian ectopic pregnancy. Hence, a beta-human chorionic gonadotropin test was done to rule out ectopic pregnancy. The patient underwent exploratory laparotomy as a conclusive diagnosis could not be reached. Histological examination finally revealed ovarian oedema.

## Introduction

Massive oedema of the ovary is a rare clinical entity. Most commonly, young women are affected [1]. Massive oedema can involve one or both ovaries. It was first described by Kalstone et al. in 1969 [2]. Though the aetiology is unclear, it is suggested that the massive oedema of the ovary results from interference of venous and lymphatic flow due to partial or complete torsion of the mesovarium [1]. It is important to diagnose this condition as it is usually misdiagnosed as malignancy, and young women undergo aggressive treatment, which results in loss of hormonal function and infertility [1]. Only very few cases of massive ovarian oedema have been published from the Indian population.

This case report was presented as a poster at the Karnataka State Obstetrics & Gynaecology Association in September 2019.

## Case presentation

**Figure 1:**
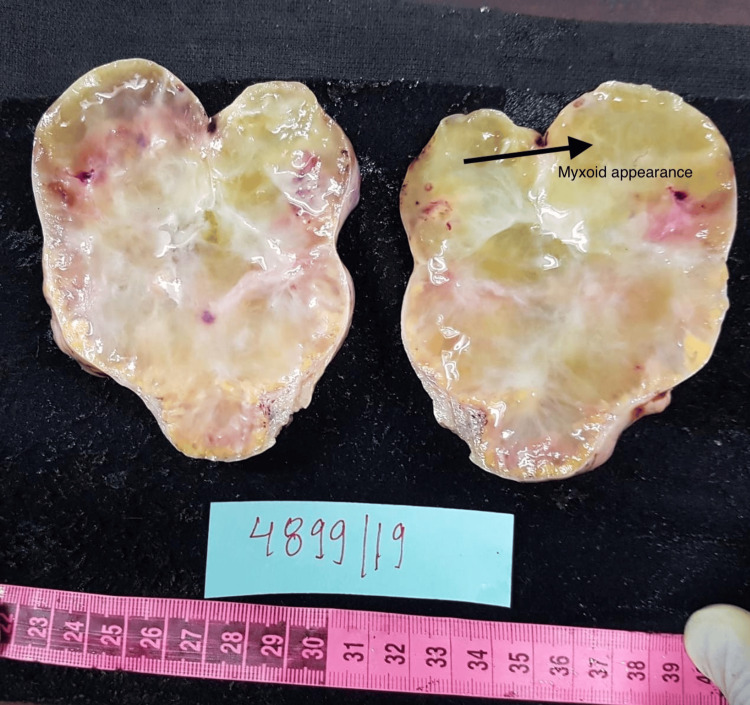
Cut surface of the ovary. Almost featureless and myxoid.

**Figure 2:**
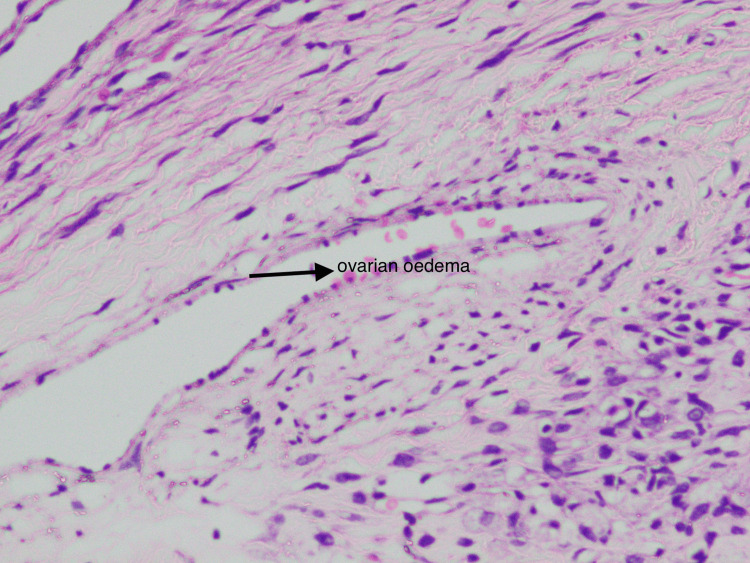
Microscopic appearance. Marked oedema and abnormally prominent vascular and lymphatic channels.

This is a case of a 27-year-old gravida 2 para 1 living 1 female at six weeks and one day of gestation, with a history of previous lower segment caesarean section. She was referred to the department in view of an ultrasonography report, showing an ovarian mass for evaluation, which was incidentally detected. The patient was asymptomatic. Clinical examination revealed an abdominal mass of 14 weeks' size of a gravid uterus, which was firm to hard in consistency. Cancer antigen 125 (CA-125) was 24.5 IU/ml. Ultrasonography done in our hospital showed features suggestive of a left ovarian mass of 8.5 x 5.2 cm with moderate ascites. Medical termination of pregnancy was done as requested by the patient. After the termination of pregnancy, a contrast-enhanced CT of the abdomen and pelvis was done, which showed a well-defined cystic lesion in the left adnexa with a thick, irregular enhancing wall and multiple intensely enhancing tortuous tufts of vessels seen from the ovarian artery and draining into the left ovarian vein, likely of old ovarian ectopic pregnancy. Hence, a beta-human chorionic gonadotropin test was done. It was 2540 IU/mL, and the repeated value after 48 hours was 999 IU/mL. A conclusive diagnosis could not be reached by clinical examination and imaging. The patient was informed about the dilemma in diagnosis. She was informed about the possibility of ovarian malignancy and the need for surgery. She was also informed about the possible impact on fertility after removing the affected ovary. She underwent exploratory laparotomy with left salpingo-oophorectomy after informed consent. The gross appearance of the specimen suggested it could be an ovarian neoplasm. On the cut surface, a gelatinous, homogenous soft surface with yellowish areas was noted (Figure 1). Histopathological examination showed marked oedema of the stroma, which appeared to be pale with prominent vascular channels in the deeper cortex, and medulla was present with clusters of luteinized cells in the periphery (Figure 2). Histological analysis finally revealed it to be a massive oedema of the left ovary.

## Discussion

Massive ovarian oedema is a rare clinical entity exhibiting gross enlargement of one or both ovaries as a result of the accumulation of oedema fluid in the stroma, causing separation of follicular structures [3]. Venous and lymphatic drainage is compromised, but it does not cause ischemic necrosis as the arterial supply is unaffected [3]. It is most commonly seen in women in their second and third decades. The most common presentation is acute abdomen [3], whereas, in our case, the patient was asymptomatic. Other common presentations could be abdominal distension, menstrual irregularities, and infertility [4]. Contrast-enhanced CT done in our hospital showed features suspicious of an old ectopic, which has not been seen in any of the articles reviewed. That gave us an inconclusive diagnosis. There is stromal luteinization, which can lead to masculinization in adult females and precocious puberty in prepubertal girls [5]. Kalstone et al. suggested that luteinization could be due to stretching of the stroma by the fluid oedema, which acts as a mechanical stimulus [2]. On gross appearance, an enlarged ovary with a soft, pearly external surface can be seen. Cut surface is almost featureless and myxoid, which was similar to our case [5]. The typical histological features are diffuse oedema of the medulla and inner cortex, which were seen in our case as well [5]. Superficial cortex and tunica albuginea are relatively spared. Abnormally prominent vascular and lymphatic channels are present [5], which is similar to our case. Foci of fibromatosis have been seen in massive ovarian oedema in some studies [6]. An intra-operative frozen section is always valuable at the time of surgery, as it can assist in performing fertility sparing surgery [1]. Intraoperative frozen section was not performed in our case. Unilateral salpingo-oophorectomy has been traditionally done in massive ovarian oedema. It could be due to failure to consider the diagnosis preoperatively [5]. This was the same with our case as well. An extensive study done by Praveen et al. identified that the majority of the patients have been over-treated with salpingo-oophorectomy due to a mistaken diagnosis of malignancy [7]. When the condition of ovarian oedema is suspected, wedge resection with the removal of 30% or more tissue is the appropriate treatment [4]. Although wedge resection may negatively affect fertility due to adhesion formation, it is a better management option than the removal of ovaries [7]. A study done by Cheng et al. reported that with detorsion, wedge resection, and plication of the ovary, the patient was successfully relieved of abdominal pain and experienced no recurrence during the follow-up period [4]. Praveen et al. performed intraoperative frozen section. Once the diagnosis of massive ovarian oedema was made, an ovarian decompression by ovarian drilling, followed by wedge resection and reconstruction of the distorted ovary with redundant capsule close to normal, was performed with great success [7].

## Conclusions

This case report illustrates the challenges in diagnosing ovarian oedema. Though a rare clinical entity, massive oedema of the ovary should be suspected in young individuals presenting with an ovarian mass. It should be considered as a differential diagnosis to avoid aggressive treatment in young patients. Intraoperative frozen section can play a pivotal role in diagnosis and conservative management. Conservative options like wedge resection with fixation of the ovary would be more appropriate, as it preserves ovarian function and fertility.
